# Curcumin alleviates 1-methyl- 4-phenyl- 1,2,3,6-tetrahydropyridine- induced Parkinson’s disease in mice via modulating gut microbiota and short-chain fatty acids

**DOI:** 10.3389/fphar.2023.1198335

**Published:** 2023-06-14

**Authors:** Benchi Cai, Lifan Zhong, Qitong Wang, Wendi Xu, Xi Li, Tao Chen

**Affiliations:** Affiliation Hainan General Hospital, Hainan Affiliated Hospital of Hainan Medical University, Haikou, Hainan, China

**Keywords:** curcumin, Parkinson disease, gut microbiota, short-chain fatty acids (SCFAs), gut-brain axis

## Abstract

**Background:** The microbiota–gut–brain axis has been proposed as a potential therapeutic target of PD. The effects of curcumin against Parkinson’s disease have been demonstrated; however, its neuroprotective mechanisms remain unknown. Our study investigated the potential mechanisms through which curcumin ameliorates Parkinson’s disease via the microbiota–gut–brain axis.

**Methods:** Mice were randomly divided into four groups: control, Curcumin, MPTP, and MPTP + Curcumin. Motor deficits and gastrointestinal dysfunction were assessed using behavioral test, intestinal motility test, and fecal parameter measurement. The loss of dopaminergic neurons and intestinal barrier function was measured using Western blot and immunofluorescence. Shotgun metagenomic sequencing and LC-MS were parallelly performed on mice feces to investigate alterations in microbiota and metabolites.

**Results:** Curcumin alleviated motor deficits and the loss of dopaminergic neurons in MPTP-induced mice. Curcumin ameliorated gastrointestinal and intestinal barrier dysfunctions in MPTP-induced mice. Curcumin reduced gut microbial dysbiosis and modulated carbohydrate metabolism in MPTP-induced mice. Curcumin restored short-chain fatty acid (SCFA) profiles in MPTP-induced mice.

**Conclusion:** Concurrently, these results indicate that curcumin inhibits Parkinson’s disease by regulating the gut microbiota and short-chain fatty acids.

## 1 Introduction

The loss of dopaminergic neurons in the substantia nigra and abnormal protein aggregation of-synuclein are characteristic of Parkinson’s disease (PD), a progressive neurodegenerative disease accompanied by symptoms such as bradykinesia, tremors, and rigidity (Tolosa et al., 2021). As the population is aging, PD imposes a major burden on society affecting more than 1% of the population over the age of 60 years (Breasail et al., 2022). However, the pathogenesis of PD is mostly unknown. It might begin in the altered gut microbiota (Sampson et al., 2016), and the gut–brain axis (Perez-Pardo et al., 2019) has recently been implicated in PD.

The human gut microbiota impacts on host wellbeing and is a large and diverse microbial community in the human gastrointestinal tract. The gut microbiota modulates many diseases through various mechanisms related to metabolism, immunity, inflammation, and intestinal epithelial barrier integrity. The association of the gut microbiota with Parkinson’s disease has been reported, although the specific components of the microbiota involved in the development of PD remain unknown (Gubert et al., 2020; Pisa et al., 2020). An increasing number of studies have confirmed the effects of different microbiota with the same metabolic functions on PD, particularly short-chain fatty acid (SCFA)-related microbiota (Meng et al., 2019). SCFAs, including acetic, propionic, and butyric acids, are produced through bacterial fermentation of non-digestible carbohydrates (Rauf et al., 2022). The benefits of SCFAs are increasingly being appreciated, with a major focus on the gut–brain axis (Dalile et al., 2019). A series of studies have measured fecal concentrations of SCFA as a substitute for determining SCFA production. Fecal SCFA levels are reduced in patients with PD. Dysbiosis of the gut microbiota and altered SCFAs have been implicated in the pathogenesis of PD; however, the underlying mechanisms have not yet been elucidated (Voigt et al., 2022). In summary, SCFA may be potential therapeutic targets in PD.

**FIGURE 1:**
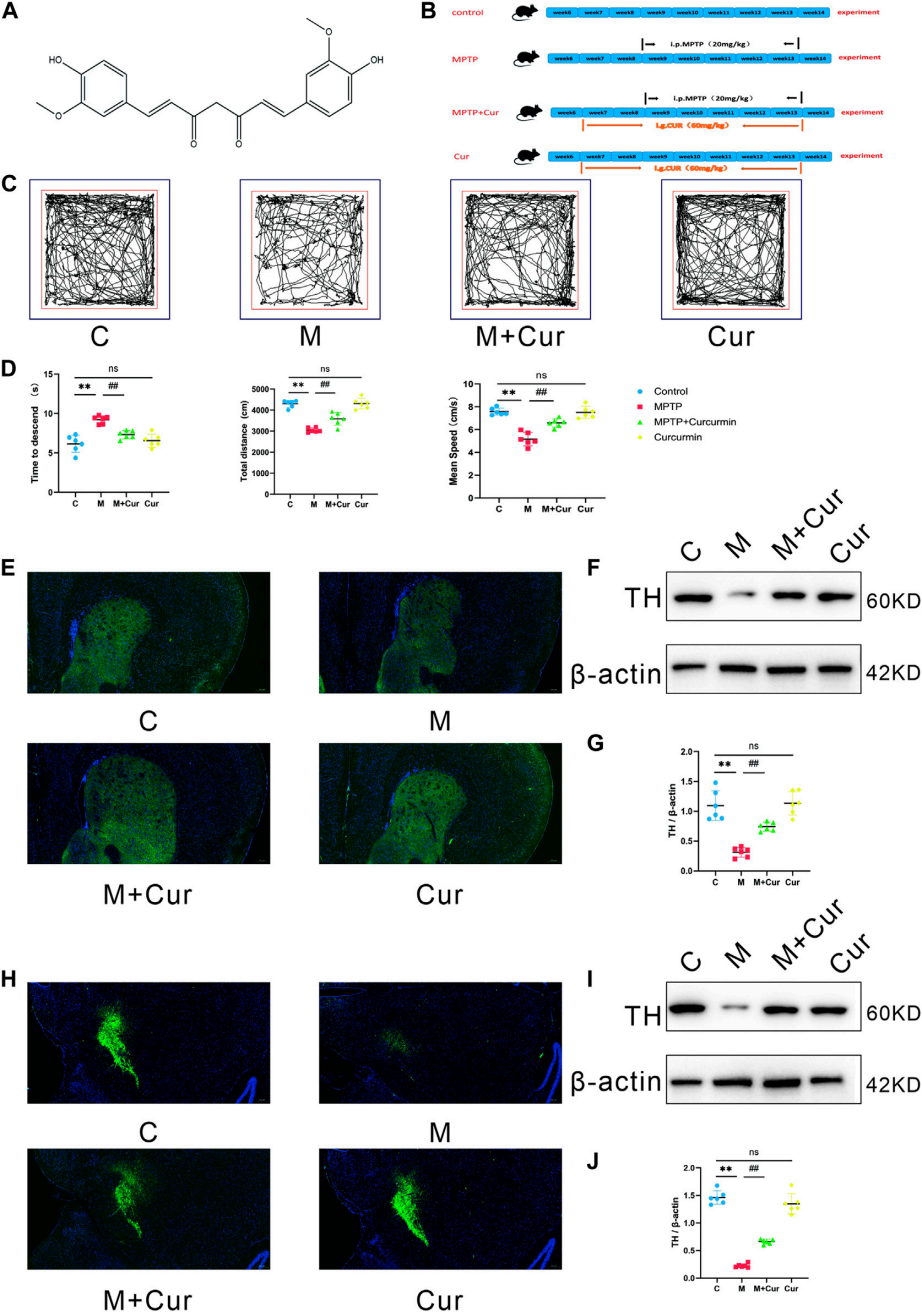
Curcumin ameliorated MPTP-induced motor deficits in PD mice. **(A)** Molecular structure of curcumin; **(B)** diagram of the experimental design; **(C)** representative traces of the open field test at 10 min; **(D)** time that is required on the top to the pole in the pole test. The distances and velocities were evaluated in the open field test **(E,H)**; representative immunofluorescence images of TH-positive cells in the SN and striatum; **(F,I)** Western blot band of TH expression in the SN and striatum; **(G,J)** Western blot analysis of TH expression in the SN and striatum; data are expressed as mean ± SEM (*n* = 6). The significance is expressed as *p < 0.05, **p < 0.01, vs. control group; ^#^ *p* < 0.05, ^##^ *p* < 0.01, vs. MPTP group; NS, statistically not significant.

Curcumin (CUR) (Figure 1A) is a natural polyphenolic compound isolated from turmeric rhizomes. Curcumin has several pharmacological properties, including antioxidant, anti-inflammatory, and anticancer (Fu et al., 2021). The effects of curcumin against Parkinson’s disease have been demonstrated; however, its neuroprotective mechanisms remain unknown owing of poor blood–brain permeability and bioavailability (Nebrisi, 2021).

This study investigated the neuroprotective effect of curcumin in a 1-methyl-4-phenyl-1,2,3,6-tetrahydropyridine (MPTP)-induced mouse model of PD. These results demonstrate that CUR ameliorates motor deficits and reduces dopaminergic neuron loss. Contrarily, curcumin ameliorated gastrointestinal and intestinal barrier dysfunctions in MPTP-induced mice. Furthermore, we analyzed the concentrations and composition of the gut microbiota and SCFAs to explore the potential mechanisms. These findings suggest that the neuroprotective role of CUR in PD is associated with CUR-induced changes in the intestinal microbiota and altered levels and composition of SCFAs.

## 2 Materials and methods

### Animals and treatments

Eight-week-old male mice of the C57BL/6J strain were procured from Hunan Slaughter Jingda Laboratory Animal Co., Ltd. (SCXK (Xiang) 2019-0004) (*n* = 24). The mice were kept in a standard environment for a week before the experiments, with free access to food and water, and under pathogen-free conditions, with a temperature of 24°C ± 2°C, 55% ± 10% humidity, and a 12 h/12 h light/dark cycle. Animal procedures were conducted in compliance with the National Institutes of Health Guide for the Care and Use of Laboratory Animals (Guide No. 55 issued by the Ministry of Health, revised edition 1998) and approved by the Ethics Committee of Hainan Hospital, Hainan Medical College (approval number [2022] 203, 22/04/2022). All mice were euthanized at humane endpoints using carbon dioxide asphyxiation. They were randomly divided into four groups (*n* = 6): control, Curcumin, MPTP, and MPTP + Curcumin (treatment group). MPTP (Sigma-Aldrich, United States) was used to induce PD in a mouse model. The model mice were given intraperitoneal injections of MPTP (20 mg/kg in saline) and probenecid (250 mg/kg in tris-HCl buffer) every 3.5 days injection for 5 weeks, for a total of 10 doses (Meredith et al., 2008; Meredith and Rademacher, 2011). Starting on day 1 of the experiment, curcumin (60 mg/kg, i.g.) (Macklin, Shanghai, China) was administered daily through gavage for 7 weeks in the Curcumin and MPTP + Curcumin groups (Figure 1B).

### Open field test

The open field test involved placing the mice in a 45 × 45 × 50-cm white square field and recording their behavior for 10 min using a Sony video camera. The results, including the trajectory, total distance, and average speed of the mice, were recorded and analyzed using the SMART 3.0 system. The field was cleaned using 70% alcohol before each use (Zhong et al., 2022).

### Pole test

The pole test was conducted based on a behavioral protocol, with mice acclimatized to their environment for 3 days before the test. Mice were placed head-up on a rough-surfaced pole during the test and timed while climbing down. The mice were required to retest if they stopped midway or climbed up again. All mice were formally tested after 5 days of training, and the average time was analyzed for motor function (Zhong et al., 2022).

### Fecal parameter measurement

The fecal parameter measurement involved placing each mouse in a clean cage wiped with 75% alcohol and recording the number of fecal pellets collected at 15 min. The feces were weighed to obtain wet weight, dried at 50°C for 24 h, and weighed again to acquire dry weight. The fecal water content was calculated using a formula: water content of feces = dry weight of feces/wet weight of feces × 100% (Zhu et al., 2017; van de Wouw et al., 2020).

### Intestinal motility test

The intestinal motility test used FITC-labeled dextran with a molecular mass of 4 kDa (FD4) to assess intestinal mobility and permeability. The mice were administered a solution of FD4 (Sigma-Aldrich, United States) at a dosage of 200 mg/kg through gavage after 6 h of food fasting. The mice were anesthetized with isoflurane and euthanized 3 h later. The entire digestive tract of the mice was extracted from the cardia to the rectum. The digestive tract was observed using an IVIS Spectrum BL small-animal optical imaging system (PerkinElmer, United States) at an excitation wavelength of 488 nm and an emission wavelength of 520 nm (Singh et al., 2016; Chen et al., 2019).

### Immunofluorescence

The mice were euthanized, and their bodies were infused with a 4% paraformaldehyde solution after the behavioral tests. The brain and cecum were removed and soaked in a 30% sucrose solution to remove any excess water. The samples were then embedded in OCT and cut into sections that were 25 µm thick. The sections were blocked and treated with primary antibodies, followed by fluorescent or horseradish peroxidase (HPR)-labeled secondary antibodies. The antibodies used were anti-TH, anti-ZO-1, anti-occludin, and Cy3-conjugated donkey anti-rabbit Ig, which were all obtained from Servicebio (Wuhan, China).

### Western blotting

Tissues were homogenized using RIPA lysis solution, and the homogenate was placed on ice for 30 min before centrifuging at 2,000 rpm and 4°C for 10 min. The resulting supernatant was used as the total protein solution. Protein concentration was measured using the BCA Protein Concentration Assay Kit (Beyotime, Shanghai, China) based on the manufacturer’s instructions. The protein solution was mixed with protein loading buffer at a ratio of 4:1 and denatured in boiling water for 15 min. The total protein in each sample was electrophoresed using 10% SDS-PAGE with PVDF (0.22 µm) membrane transfer. The membranes were blocked with 5% skimmed milk powder, incubated with primary antibody overnight at 4°C, and then incubated with secondary HPR antibody. The protein bands were detected using Super Enhanced Chemiluminescence (ECL) detection reagent (Bio-Rad, CA, United States) and a gel imaging system (Tanon, Shanghai, China). The band intensity was quantified using ImageJ software (version 1.8.0, NIH). Anti-TH antibody (Servicebio, Wuhan, China) was used as the primary antibody.

### Whole-metagenome shotgun total

DNA from the samples were extracted using the QIAamp Fast DNA Stool Mini Kit (Qiagen, Hilden, Germany) following the manufacturer’s instructions. The DNA concentration and integrity were assessed using a NanoDrop2000 spectrophotometer (Thermo Fisher Scientific, Waltham, MA, United States) and agarose gel electrophoresis, respectively. DNA was fragmented using an S220 focused ultrasonicator (Covaris, United States) and cleaned using Agencourt AMPure XP beads (Beckman Coulter Co., United States). The TruSeq Nano DNA LT Sample Preparation Kit (Illumina, San Diego, CA, United States) was used to construct libraries following the manufacturer’s instructions. Shotgun metagenomic sequencing was performed using the Illumina NovaSeq 6000 platform. The sequences in the FastQ file were trimmed and filtered using Trimmomatic (v 0.36) (Bolger et al., 2014). Abundance statistics were performed at each level of Domain, Kingdom, Phylum, Class, Order, Family, Genus, and Species to construct the abundance profile at the corresponding taxonomic level. The representative gene set sequence was annotated with KEGG database (Kanehisa et al., 2014) with an e-value of 1e−5 using DIAMOND (v 0.9.7) (Buchfink et al., 2015). Information was obtained on the carbohydrate active enzyme corresponding to the gene using the CAZy database, and carbohydrate activity was calculated using the sum of the gene abundances corresponding to the carbohydrate active enzyme abundance (Cantarel et al., 2009). The taxonomic or functional abundance spectra was PCoA analyzed and plotted using the R software (v 3.2.0). Significant differences between groups were analyzed using t-test/Wilcoxon statistical tests. The taxonomic and functional abundance spectra were compared using the linear discriminant analysis effect size (LEfSe) method. Metagenome sequencing and analyses were conducted by OE Biotech Co., Ltd. (Shanghai, China).

### UPLC-MS/MS

SCFAs were measured using LC-MS. For derivatization 80 μL of each standard solution or of each of the supernatants were mixed with 40 μL of 200 mM 3NPH in 50% aqueous acetonitrile and 40 μL of 120 mM EDC-6% pyridine solution in the same solvent. The mixture was reacted at 40°C for 30 min. Afterward samples were placed at −20°C for 30 min, and thenfiltered through a 0.22 μm organic phase pinhole filter for subsequent UPLC-MS/MS analysis. Liquid chromatography was performed using the Nexera UHPLC LC-30A (SHIMADZU). ACQUITY UPLC BEH C18 (100*2.1 mm, 1.7 μm) was used for analysis. Mobile phase A consisted of water containing 0.1% formic acid, and the mobile phase B was acetonitrile B. The LC gradient was 0 min, 10% B; 1 min, 10% B; 2 min, 25% B; 6 min, 35% B; 6.5 min, 95% B; 7.8 min, 95% B; and 8.5 min, 10% B. All the samples were kept at 4°C during the analysis, the column temperature was set at 40°C, and injection volume was 5 μL. Mass spectrometry was performed on an AB SCIEX Selex ION Triple Quad™ 5500 System operating in both positive and negative ion modes. Nitrogen was used as collision gas. Targeted metabolites were analyzed in multiple reaction monitoring (MRM) mode. MRM pairs, declustering potentials (DP), and collision energies (CE) were optimized for each analyte. Data acquisition and further analysis were conducted using the Analyst software. The proportional distribution of individual SCFA to total SCFA was calculated using the SCIEX OS-MQ software. This experiment and the analyses were conducted by Luming Biotech Co., Ltd. (Shanghai, China).

### Correlation analysis of the microbiome and metabolome

Spearman’s rank correlation coefficient was calculated for the gene function of metagenome–metabolite and metabolite–behavioral indicator pairs using data across the same samples. These relationships were visualized with Sankey using NetworkD3 (version 0.4) in the R package.

### Statistical analysis

The data were expressed using mean ± SEM, and statistical differences between groups were indicated using *p*-values, with a significance level of *p* < 0.05. All results were analyzed using the Kruskal–Wallis test through the GraphPad Prism 6 software.

## 3 Result

### Curcumin ameliorated motor deficits in MPTP-induced mice

Behavioral tests were administered to examine the effects of curcumin in MPTP-induced motor deficits. Particularly, we used the open-field and pole tests. The locomotor tracks of different groups of mice in the open field test were recorded for 10 min (Figure 1C). The MPTP group displayed a significant reduction in the total movement distance (*p* < 0.01) and mean speed (*p* < 0.01) compared with the control group. The MPTP + Curcumin group displayed a significant increase in the total movement distance (*p* < 0.01) and mean speed (*p* < 0.01) compared with the MPTP group. Differences between the control and Curcumin groups were not statistically significant (Figure 1D).

The mice were subjected to the pole test in addition to evaluation in the open field. The time required to descend the pole was measured using a stopwatch and recorded. MPTP treatment significantly extended the time to descend the pole compared with that in the control group (*p* < 0.01), and MPTP + Curcumin group displayed a significant decrease compare with MPTP group (*p* < 0.01), whereas no statistical difference was noted between the Curcumin and control groups (Figure 1D).

Behavioral test assay results showed that the MPTP + Cur group had a longer distance of locomotion and higher velocity in the open field test and a shorter time to descend in the pole test, compared with the MPTP group. The administration of curcumin significantly rescued these reductions in locomotor activities in MPTP-treated mice (Figures 1C, D).

### Curcumin reduces MPTP-induced loss of dopaminergic neurons

Tyrosine hydroxylase (TH), the rate-limiting enzyme responsible for dopamine synthesis, is used as a marker of dopaminergic neuronal integrity in Parkinson’s disease models. The expression of TH proteins in the SN and striatum was evaluated using Western blot and immunofluorescence.

Figures 1F, I display representative results of Western blot analysis. The results indicated that the MPTP group had significantly lower levels of TH expression in the SN (*p* < 0.01) and striatum (*p* < 0.01) than the control group, whereas no significant difference was observed between the Curcumin and control groups. However, the MPTP + Cur group showed a significant improvement in TH expression in the SN (*p* < 0.01) and striatum (*p* < 0.01) compared with the MPTP group (Figures 1G, J). The immunofluorescence staining results agrees with the Western blotting results (Figures 1E, H). These data support Curcumin reduces MPTP-induced loss of dopaminergic neurons.

### Curcumin ameliorates gastrointestinal dysfunction in MPTP-induced mice

**FIGURE 2:**
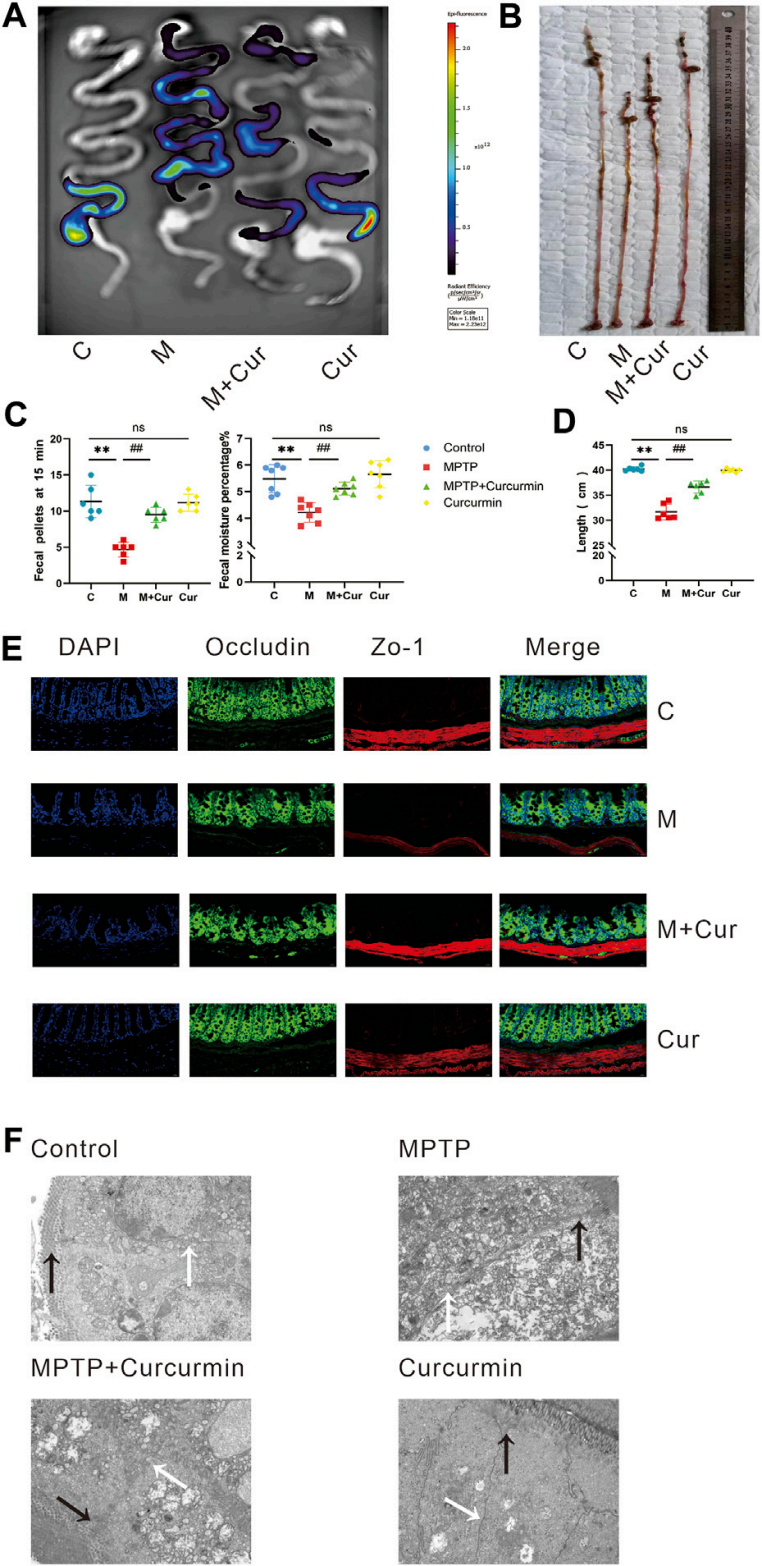
Curcumin ameliorated MPTP-induced gastrointestinal dysfunction in PD mice. **(A)** The representative fluorescent imaging for assessing intestinal motility were visualized; **(B)** representative of the gastrointestinal tract; **(C)** fecal water content; number of 15-min fecal pellets in mice; **(D)** length of gastrointestinal tract; **(E)** representative immunofluorescence images of zo-1 and occludin-positive cells in the intestine; **(F)** intestinal barrier was observed using transmission electron microscopy (tight junctions labeled black arrow and the gap junctions labeled white arrow); data are expressed as mean ± SEM (*n* = 6). The significance is expressed as *p < 0.05, **p < 0.01, vs. control group; ^#^ *p* < 0.05, ^##^ *p* < 0.01, vs. MPTP group; NS, statistically not significant.

A gastrointestinal dynamics assay was conducted to evaluate the potential of curcumin in alleviating gastrointestinal dysfunction in MPTP-induced PD mice. The results presented in Figure 2A are representative images of the different groups. The strongest fluorescence signals in the colon were observed in the control and Curcumin groups, whereas the strongest fluorescence signals in the upper and lower portions of the small intestine were observed in the MPTP and MPTP + Cur groups, respectively. The above results suggest that curcumin can reduce MPTP-induced gastrointestinal motor dysfunction.

MPTP-induced mice exhibited shortened intestinal length compared to the control group (*p* < 0.01), whereas the Curcumin group showed no significant difference in intestinal length compared with the control group (*p* > 0.05). However, the MPTP + Cur group showed significantly improved intestinal length compared with the MPTP group (*p* < 0.01) (Figures 2B, D). And such change of MPTP induced gastrointestinal shortening could be rescued by curcumin therapy.

Constipation is a common gastrointestinal dysfunction in PD, and fecal parameters were measured to assess this symptom in MPTP-induced mice. The results showed that the fecal moisture content and number of 1-min fecal pellets decreased in the MPTP group compared with those in the control group (*p* < 0.01). The Curcumin group did not show significant changes in fecal parameters compared with the control group (*p* > 0.05). The MPTP + Cur group showed a slight increase in fecal water content and the number of fecal pellets at 15 min compared with the MPTP group (*p* < 0.01) (Figure 2C). These results suggest that gastrointestinal symptoms, including constipation, improved significantly.

### Curcumin ameliorates intestinal barrier dysfunction in MPTP-induced mice

Immunofluorescence staining of two tight junction proteins, ZO-1 and occludin, was performed to assess whether MPTP-induced barrier disruption in the intestines was ameliorated by curcumin. Tight (TJs) and gap junctions (GJs) between gut epithelial cells were examined using a transmission electron microscope. The results showed that TJs (black arrow) were disrupted and GJs (white arrow) were indistinct in MPTP-induced mice, accompanied by a reduction in occludin and ZO-1, whereas no significant change was noted in immunofluorescence and transmission electron microscopy in the MPTP + Cur group compared with the control or Curcumin group (Figures 2E, F). These results suggest that curcumin can ameliorates intestinal barrier dysfunction in MPTP-induced mice.

### Curcumin recovers gut microbial dysbiosis in MPTP-induced mice

**FIGURE 3:**
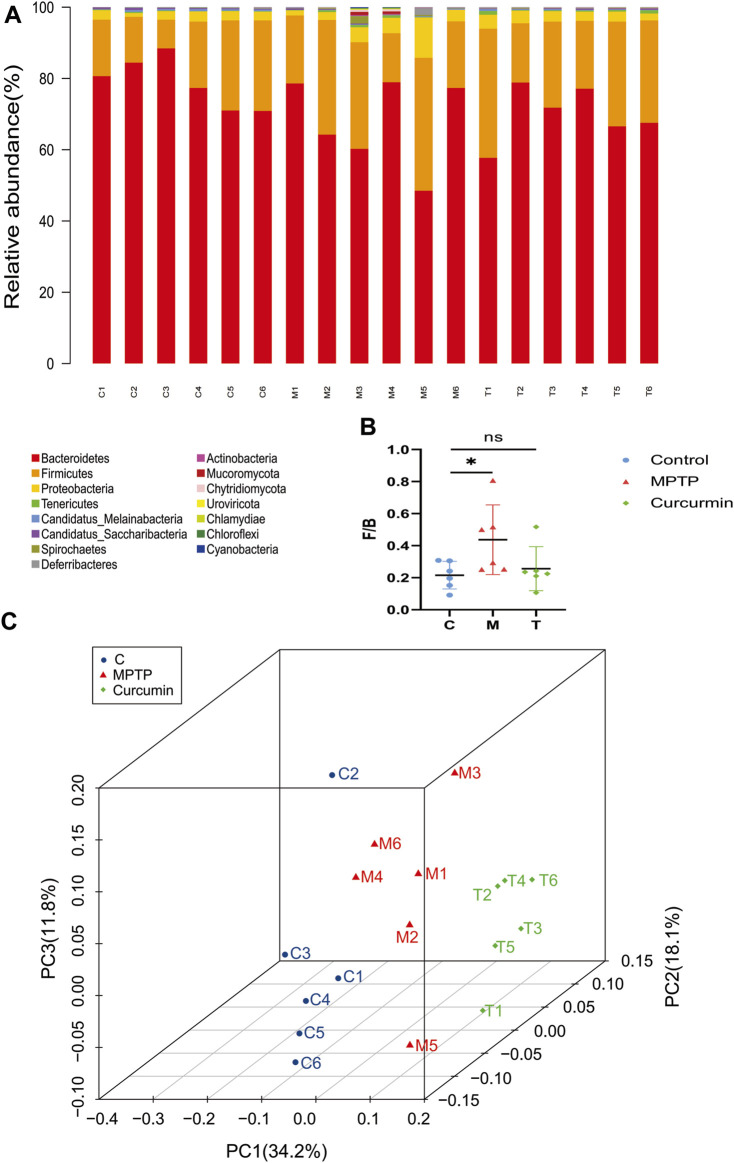
Curcumin recover gut microbial dysbiosis in the phylum level. **(A)** Stacked bar chart indicating the proportions of gut microbiota in the levels of phylum, and different colors represent different species; **(B)** Firmicutes/Bacteroidetes ratio; **(C)** distance metric was ordinated into 3D via PCoA and visualized; data are expressed as mean ± SEM (*n* = 6). The significance is expressed as *p < 0.05, **p < 0.01, vs. control group; ^#^ *p* < 0.05, ^##^ *p* < 0.01, vs. MPTP group; NS, statistically not significant; Treatment, MPTP + Curcumin group.

We performed a microbiota analysis using whole-genome shotgun sequencing to investigate whether curcumin treatment affected the gut microbiota of PD mice. The Firmicutes/Bacteroidetes (F/B) ratio influences the maintenance of normal intestinal homeostasis at the phylum level. The species composition and F/B ratio results suggest that gut microbial dysbiosis occurred in MPTP-induced mice (*p* < 0.05) (Figures 3A, B). The F/B ratio decreased slightly although no statistical difference was noted in the MPTP + Cur group (treatment group) compared with the MPTP group (Figures 3A, B), which means curcumin can recover gut microbial dysbiosis in MPTP-induced mice.

**FIGURE 4:**
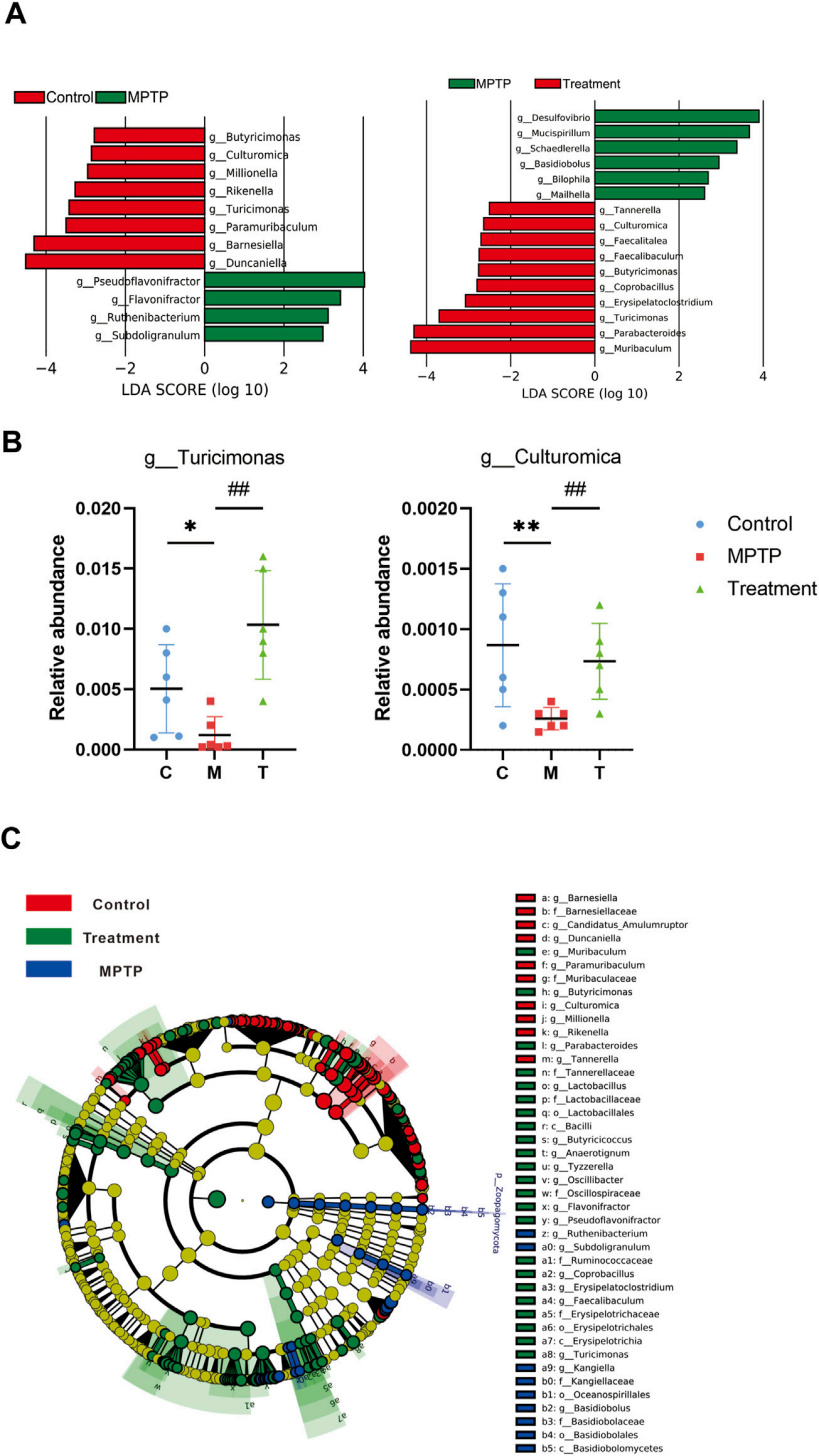
Curcumin recover gut microbial dysbiosis in the genus level. **(A)** LDA scores of altered taxa in the levels of genus [LDA scores (log10) >2 are listed]; **(B)** relative abundance of turicimonas and culturomica; **(C)** Grouping was made for each variable based on the Evolutionary Tree. Data are expressed as mean ± SEM (*n* = 6). The significance is expressed as *p < 0.05, **p < 0.01, vs. control group; ^#^ *p* < 0.05, ^##^ *p* < 0.01, vs. MPTP group; Treatment, MPTP + Curcumin group.

Principal coordinate analysis (PCoA) based on the relative abundance at the species level (Figure 3C) revealed a significant separation in bacterial community composition among the control, MPTP, and MPTP + Cur (treatment) groups using the first three principal component scores of PC1, PC2, and PC3 (34.2%, 18.1%, and 11.8% of explained variance, respectively). Notably, alterations with LDA scores of >2 were identified across the three groups at the genus (Supplementary Material S1) and species (Supplementary Material S2) levels. Linear discriminant analysis (LDA) coupled with effect size measurements showed that 8 bacterial taxa were enriched in the control group, 4 in the MPTP group, and 14 in the MPTP + Cur group (treatment group) (Figure 4A). Paramuribaculum, Barnesiella, and Duncaniell were dominant in the MPTP group compared with the control group, whereas Pseudobflavonifractor, Flavonifractor, and Ruthenibacterium were less abundant. Turicimonas, Parabacteroides, and Muribaculum were dominant in the MPTP + Cur group (treatment group) compared with the MPTP group, whereas Desulfovibrio, Mucispirillum, and Schaedlerella were less abundant. The MPTP + Cur group (treatment group) exhibited an increase in Turicimonas (*p* < 0.01) and Culturomica (*p* < 0.01) indicating the potential restoration of the normal microbiota with treatment (Figure 4B). Interestingly, similar results were obtained in the evolutionary trees of these species, which was constructed as in Figure 4C. These suggests that curcumin can restore MPTP-induced microbial dysbiosis to normal composition in part, with Turicimonas and Culturomica playing a major role.

### Curcumin modulates carbohydrate metabolism in MPTP-induced mice

**FIGURE 5:**
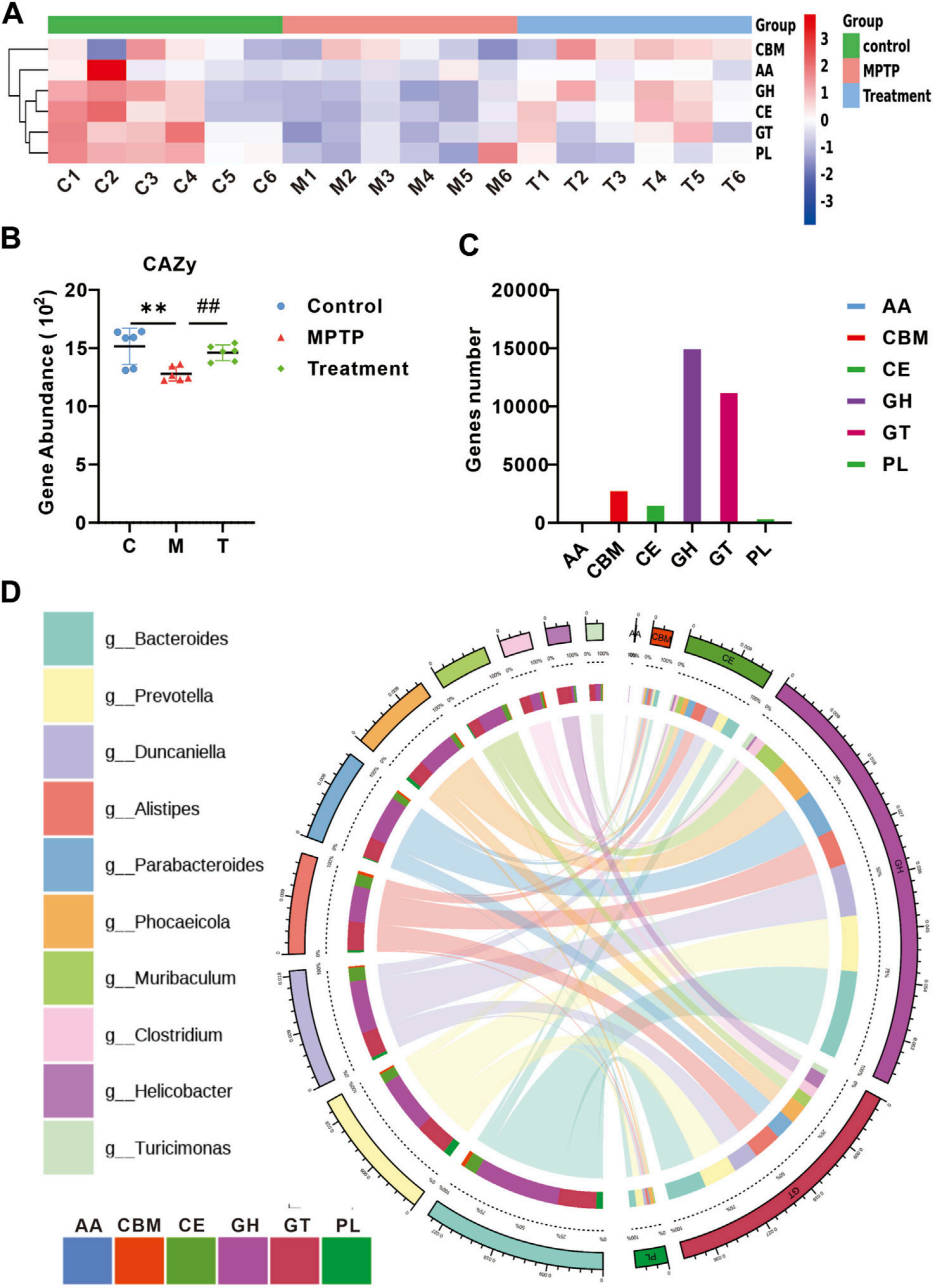
Curcumin modulates carbohydrate metabolism in PD mice. **(A)** Heat map showing the abundance of altered CAZy family in each group; **(B)** relative gene abundance of carbohydrate-degrading enzymes (CAZy); **(C)** curcumin-associated genes were classified into enzyme families according to the CAZy function; **(D)** Circo plots indicating the relationship between the top 10 abundant bacteria and enzyme families in the treatment group; data are expressed as mean ± SEM (*n* = 6). The significance is expressed as *p < 0.05, **p < 0.01, vs. control group; ^#^ *p* < 0.05, ^##^ *p* < 0.01, vs. MPTP group; GH, glycoside hydrolases; GT, glycosyl transferases;PL, polysaccharide lyases; CE, carbohydrate esterases; AA, auxiliary activities; CBM, carbohydrate-binding modules; Treatment, MPTP + Curcumin group.

Functional analyses were performed using the Carbohydrate-Active enZymes database (CAZy). The CAZy database describes the families of carbohydrate metabolism-related enzymes. A heat map depicts the differences between each CAZy family in each group (Figure 5A). Gene abundance analysis of CAZy showed the restoration of carbohydrate metabolic functions in MPTP + Cur group (treatment group) (Figure 5B), particularly glycoside hydrolases (*p* < 0.01) and glycosyl transferases (*p* < 0.01) (Figure 5C). Circos plots showed that Turicimonas, Parabacteroides, and Muribaculum, which are dominant in the treatment group, explained 21.2% of the glycoside hydrolases and 18.4% of glycosyltransferases (Figure 5D). These results indicate that curcumin modulates carbohydrate metabolism in MPTP-induced mice.

### Curcumin restores short-chain fatty acid (SCFA) profiles in MPTP-induced mice

**FIGURE 6:**
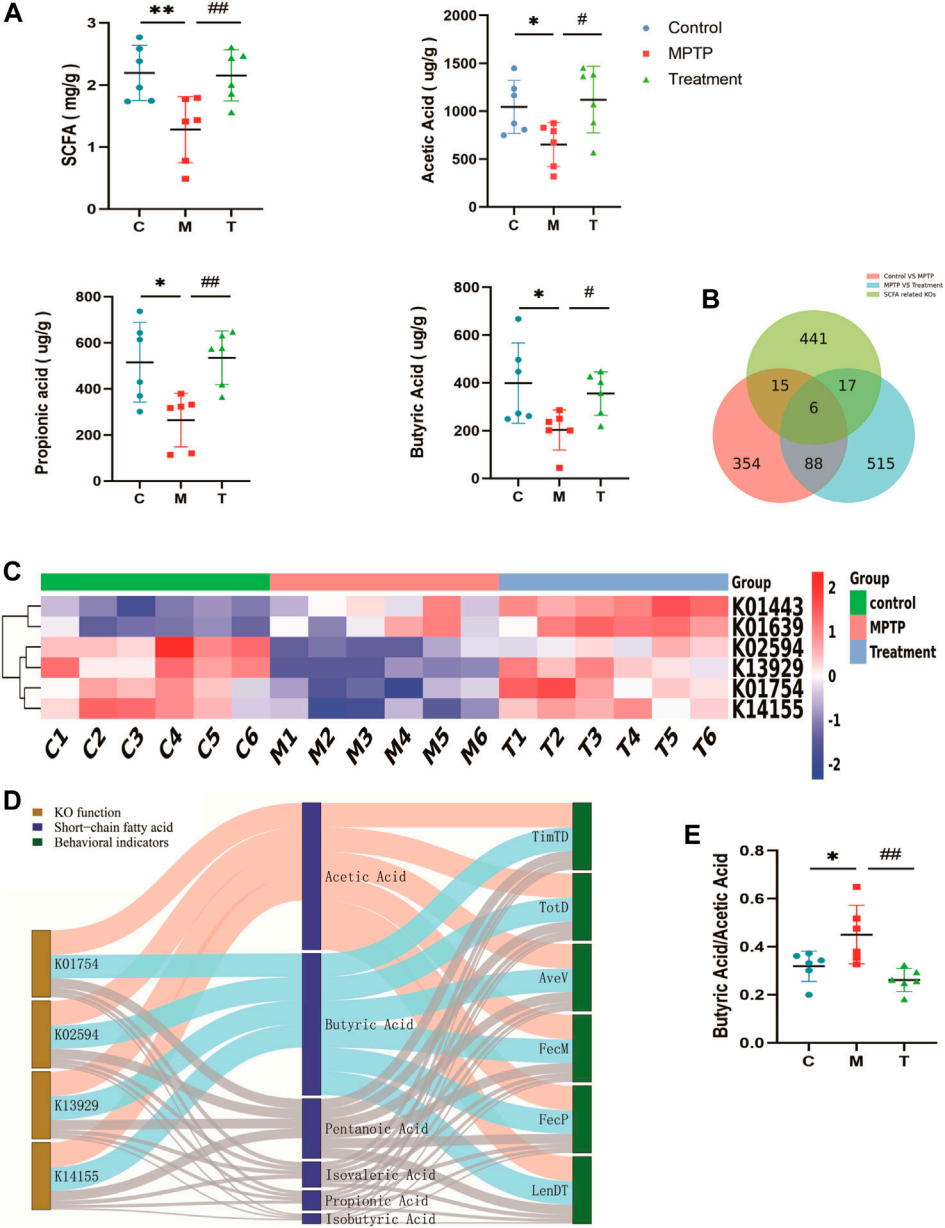
Curcumin restores short-chain fatty acid (SCFA) profiles in PD mice. **(A)** Individual concentrations of total short-chain fatty acid and each composition; **(B)** Venn diagram showing the number of KOs that overlap with three groups; Red The differential KOs between the control and MPTP groups compared; Blue The differential KOs between the Treatment and MPTP groups compared; Green SCFA related KOs; **(C)** The heat map of overlap KOs for Venn diagram; **(D)** Sankey Network diagram depicting the information flow among key KOs, SCFAs and behavioral indicators of mice;Blue indicates negative correlation (Spearman correlation ≤ −0.8, *p* < 0.05) and red indicates positive correlation (Spearman correlation ≥ 0.8, *p* < 0.05), remaining links were represented by gray dots; **(E)** Histogram of butyrate acid/acetic acid in three groups; TimTD Time to descend (s), TotD Total distance (m), AveV Average Velocity (m/s), FecM Fecal moisture percentage %, FecP Fecal pellets at 15 min,LenDT Length (cm); Data are expressed as the mean ± SEM (*n* = 6). The significance is expressed as *p < 0.05, **p < 0.01, vs control group; #*p* < 0.05, ##*p* < 0.01, vs MPTP group;

The gut microbiota degrades carbohydrates to harvest energy and provides the host with a variety of metabolites such as short-chain fatty acids. We measured short-chain fatty acid (SCFA) profiles in mouse feces. SCFAs were depleted in MPTP-induced mice, and treatment with curcumin restored total short-chain fatty acids, acetic acid, propionic acid, and butyric acid (Figure 6A). A Venn diagram was drawn for the 354 KO differential genes between the control and MPTP groups, the 515 KOs differential genes between the MPTP and MPTP + Cur (treatment) groups, and the SCFA-related KOs (441) obtained from the KEGG database. We noted that curcumin modulated six targets of SCFA (Figure 6B). We found that KO01754 (threonine dehydratase [EC:4.3.1.19]), KO02594 (homocitrate synthase NifV [EC:2.3.3.14]), KO13929 (malonate decarboxylase α subunit [EC:2.3.1.187]), and KO14155 (cysteine-S-conjugate β-lyase [EC:4.4.1.13]) may be functional target genes of curcumin among the predicted targets in restoring SCFA profiles (Figure 6C). A Sankey diagram displayed the relationship between mouse status and their SCFA profiles altered by information from functional genes (Figure 6D). This results that Curcumin restores short-chain fatty acid (SCFA) profiles in MPTP-induced mice to normal by KO01754, KO02594, KO13929, and KO14155. Compared with the MPTP group, reflected the SCFA Profiles change, the butyrate acid/acetic acid ratio decreased in MPTP + Cur group (treatment group) (Figure 6E).

## 4 Discussion

The pathogenic mechanisms that cause neurodegeneration in PD remain unclear; however, an increasing number of studies support the potential role of the microbiota–gut–brain axis in Parkinson’s disease (PD) (Tan et al., 2022). Thus, the therapeutic basis of microbiota–gut–brain axis has been elucidated. In this study, we investigated the possible protective effects of curcumin against Parkinson’s in an MPTP-induced Parkinson’s disease model. Dyskinesia and the loss of dopaminergic neurons in an MPTP-induced Parkinson’s disease mouse model can be reversed by curcumin, and a protective effect on gut barrier function and gastrointestinal dysfunction can also be observed. Curcumin changes the gut microbiota and its metabolites, such as short-chain fatty acids (SCFAs). Sankey suggested that curcumin can exert a beneficial effect on MPTP-induced mice by altering the levels and profile of short-chain fatty acids (SCFAs) through the modulation of several key KOs.

Curcumin, a natural compound found in *Curcuma longa*, is the most dynamic component of the Zingiberaceae (Kocaadam and Sanlier, 2017). At present, a growing body of evidence suggests that the progression of Parkinson’s disease can be slowed by treatment with curcumin (van der Merwe et al., 2017; Nguyen et al., 2018; Thiruvengadam et al., 2021; Zhang et al., 2021; Donadio et al., 2022; Fikry et al., 2022). Our results are in agreement with previous experimental data. Curcumin treatment rescued the *in vivo* loss of dopaminergic neurons and reversed MPTP-induced dyskinesia in a model of PD. However, the pharmacological effects of curcumin are impeded by its pharmacokinetic properties primarily because of the difficulty in absorbing curcumin into the bloodstream and its low bioavailability (Banerjee and Chakravarty, 2015; Lopresti, 2018). Curcumin metabolism occurs rapidly in the intestine by microbiota, leaving a small quantity in other tissues, such as the brain (Tsuda, 2018; Yavarpour-Bali et al., 2019; Askarizadeh et al., 2020). The predominant route of curcumin elimination is feces, and the highest amounts of curcumin are found in the gastrointestinal tract (Liu et al., 2019). Therefore, additional mechanisms, such as the gut–brain axis, might be associated with the effects of curcumin on PD (Chetty et al., 2021; Cui et al., 2022).

Gastrointestinal symptoms, including delayed gastric emptying and constipation, may precede the motor symptoms by decades in patients with PD. The interaction between the gut and brain has been proposed as the gut–brain axis (Agirman et al., 2021). Recently, the function of the gut–brain axis was found to contribute to understanding the pathology of PD (Menozzi et al., 2021). Several studies have indicated that the gut–brain axis is a key mechanism in the pathology of PD (Klingelhoefer and Reichmann, 2015; Klann et al., 2021; Dogra et al., 2022). Some potential mechanisms are as follows (Klingelhoefer and Reichmann, 2015). One hypothesis supports the hypothesis that sporadic PD begins in the gastrointestinal tract via misfolded α-synuclein aggregation, which leads to the retrograde transport of α-synuclein via the vagus nerve from the gut to the brain. Vagotomy decreases the risk of PD, suggesting that the vagus nerve plays an important role in PD pathogenesis. In addition, intestinal barrier disruption along the gut–brain axis contributes to PD pathogenesis of the gut–brain axis (Perez-Pardo et al., 2019). In a study conducted on patients with PD and age-matched controls, tight junction protein expression was significantly decreased in patients with PD (Clairembault et al., 2015). Results of numerous animal model studies are consistent with the above results (Dong et al., 2020; Zhou et al., 2021; Xu et al., 2022), which are in line with prior evidence. Our results showed that TJs were disrupted, and GJs were indistinct in MPTP-induced mice, accompanied by a reduction in occludin and ZO-1 levels. The gastrointestinal function remained consistent with that of the intestinal barrier. Our study and previous findings suggest that the effects of curcumin against Parkinson‘s disease may depend on the gut–brain axis; however, how the gut–brain axis is regulated is unknown.

Recently, the critical role of the gut microbiota in PD pathogenesis has received increasing attention (Sampson et al., 2016; Wang et al., 2021; Claudino Dos Santos et al., 2022). The crosstalk between the brain and gut in PD is believed to be influenced by gut microbiota dysbiosis and alterations in bacterial metabolite activities (Tan et al., 2022). Our study suggests that Firmicutes increased and Bacteroidetes decreased in MPTP-induced mice, and that curcumin could reverse this change, which is consistent with previous studies. Dysbiosis increases intestinal permeability, leading to abnormal aggregation of α-synuclein fibrils. Compared with healthy controls in clinical studies, a range of gut microbiota associated with PD are potential biomarkers of PD. Twenty-two bacterial species were considered potentially significant, with 14 increasing and 8 decreasing in PD (Scheperjans et al., 2015). These bacteria are associated with Parkinson’s disease severity, non-motor symptoms, and clinical phenotypes (Scheperjans et al., 2015). Whether curcumin exerts a neuroprotective effect on certain microbiota remains unknown given the complexity of the gut microbiota and the differences in gut microbiota communities among individuals. However, we have identified many metabolites of the gut microbiota associated with the pathogenesis of PD, such as short-chain fatty acids (Voigt et al., 2022). A nonparametric meta-analysis of the intestinal microbiota in PD indicated that short-chain fatty acid (SCFA)-producing bacteria are decreased in PD (Hirayama and Ohno, 2021).

Trillions of gut microbiota contain a treasury of enzymes. These enzymes directly metabolize dietary components and drugs; thus, the same enzymes from different gut microbiota produce the same metabolites. Short-chain fatty acids (SCFAs) are one of the most important metabolic products of the gut microbiota and associated with PD (Vascellari et al., 2020; Voigt et al., 2022). The concentrations of SCFAs were significantly lower in samples of patients with PD (Jiang et al., 2023). The pro-inflammatory state in the colon of patients with PD persists due to decreased abundance of SCFA-producing colonic bacteria in colon tissues of subjects with PD, which was an important and early pathogenesis of Parkinson’s disease (Agirman et al., 2021; Zhou et al., 2021; Zhong et al., 2022; Jiang et al., 2023). The function of SCFA in Parkinson’s disease may be related to CD3^+^ T cells, TLR4^+^ cells, and depend on the gut-brain axis (Perez-Pardo et al., 2019). Decreased SCFA downregulates regulatory T cells and fails to suppress neuronal inflammation (Zhou et al., 2021). Meanwhile there is a pathological imbalance in the acetylation and deacetylation of histones in PD, histone deacetylase inhibitors are potential drugs for PD, and SCFA is a natural and effective deacetylase inhibitor (Harrison and Dexter, 2013). Including our study, the decrease in SCFAs and SCFA-producing bacteria has been widely observed in both model organisms and PD patients (Aho et al., 2021; Chen et al., 2022; Voigt et al., 2022; Lu et al., 2023). Although the mechanisms remain to be explored. Recently a Mendelian randomizationanalysis (Jiang et al., 2023) and an in-silicoComparative analysis (Chauhan et al., 2023) have once again provided confidence to scholars advancing research in this area.

Our study found decreased expression of SCFAs in MPTP-induced mice compared with controls consistent with previous studies. Concurrently, we found that the proportion of SCFAs may be key to pathogenesis, which is more important than concentration changes. Butyric acid/acetic acid increased in the feces of MPTP-induced mice compared with that in the control samples. Butyrate acid/acetate acid in feces is consistent with our finding of gut microbiota changes and altered Firmicutes/Bacteroidetes at the phylum level. Acetic and propionic acids are mainly produced by Bacteroidetes, and butyric acid is the primary product of Firmicutes. Curcumin reversed this effect in MPTP-induced mice. These results suggest that curcumin ameliorates PD symptoms by altering the range of key SCFA-related enzymes rather than a certain microbiota.

**FIGURE 7:**
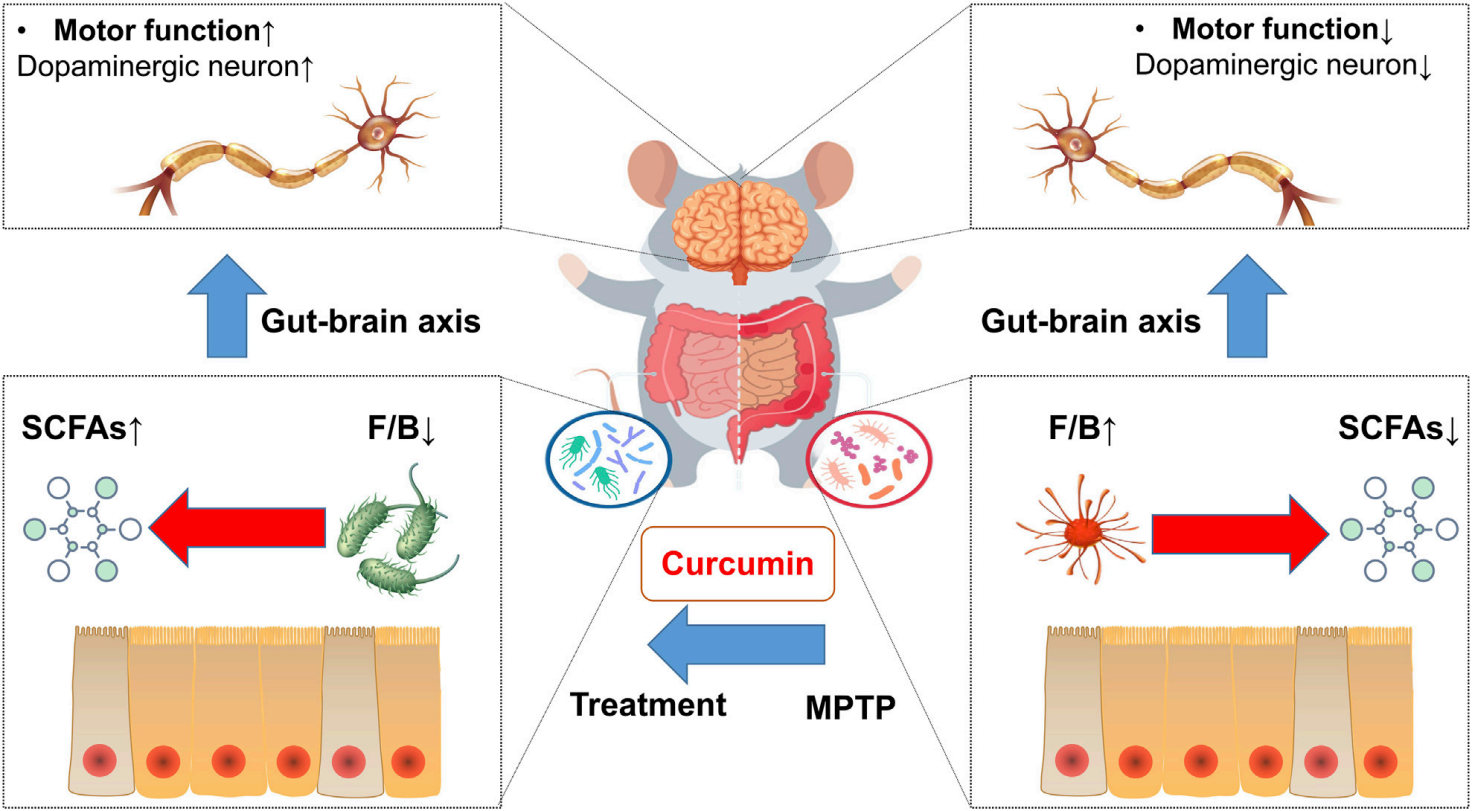
Mechanism of curcumin-modulated gut microbiota and short-chain fatty acids in MPTP-induced PD mice.

In conclusion, our study shows that curcumin inhibits Parkinson’s disease by regulating the gut microbiota and short-chain fatty acids (Figure 7). Our data show that the recovery of gastrointestinal dysfunction with curcumin ameliorates motor deficits in a mouse model of PD. Much evidence in the literature suggests that gastrointestinal dysfunction is involved in the development of PD, and our data support an important role for the gut–brain axis as described previously. In addition, SCFAs and the gut microbiota are often implicated as regulators of the gut–brain axis. However, determining whether a specific microbiota is associated with Parkinson’s disease pathogenesis is difficult because of the complexity of the intestinal microbiota. Previous studies on the relationship between SCFAs and Parkinson often focused on the concentration of SCFAs, such as butyric and acetic acids, rather than the proportion of SCFAs. Our study revealed that curcumin regulates the carbohydrate-active enzymes of the gut microbiota by directly altering the concentration and proportion of SCFAs.

These findings have significant implications for patients with PD. However we acknowledge the limitations of this study. First, the causation requires confirmation via fecal microbiota transplantation. Moreover, curcumin is metabolized by different reductases, yielding active metabolites, namely, dihydrocurcumin, tetrahydrocurcumin, and hexahydrocurcumin (Pan et al., 1999; Asai and Miyazawa, 2000; Ireson et al., 2002). This reductive metabolic reaction of curcumin occurs extensively in the intestine. Recently, 23 metabolites were registered and several novel human gut microbiota curcumin metabolic pathways, via demethylation, hydroxylation, and acetylation, or the combination of these, were revealed (Nakagawa et al., 2014). Many studies suggest the great potential of these curcumin metabolites transformed by gut microbiota as promising agents to combat neurodegenerative diseases (Lopresti, 2018). Unfortunately, due to the complexity of metabolites, the difficulty of detection and the limitations of experimental conditions, and we have not explored it in depth in this study. These effects are caused by its metabolites or curcumin itself should be further studies. Finally, to gain a better understanding, it should further investigate whether the bacteria responsible for curcumin metabolism are influenced by PD and subsequently recover after treatment. This analysis can provide insights into the potential role of gut microbiota in PD and the impact of curcumin on these specific bacterial populations. The intestinal bacterium (Blautia, *Escherichia fergusonii* ATCC 35469, *Escherichia coli* strains (ATCC 8739 and DH10B), *Bacillus megaterium* DCMB-002, *Bifidobacteria longum* BB536, *Bifidobacteria pseudocatenulatum* G4, *E. coli* K-12, *Enterococcus faecalis* JCM 5803, *Lactobacillus acidophilus*, and *Lactobacillus casei*) were involved in the metabolism of curcumin (Maehara et al., 2011; Zhang et al., 2013; Nakagawa et al., 2014; Tan et al., 2014; Burapan et al., 2017; Shen and Ji, 2019). However, to describthe specific microbial species and taxa involved in the metabolism of curcumin, only limited information is known so far (Shen and Ji, 2019). We will try to investigate this in our subsequently study and expand the sample size to further validate our results. Based on these, the altered proportion of SCFAs, the potential role of gut microbiota in PD, the impact of curcumin and its metabolites on these specific bacterial populations should be the focus of further studies on curcumin alleviation in PD.

## Data Availability

The datasets presented in this study can be found in online repositories. The names of the repository/repositories and accession number(s) can be found in the article/Supplementary Material.
