# Supplementary figures and images for: Curcumin alleviates 1-methyl- 4-phenyl- 1,2,3,6-tetrahydropyridine- induced Parkinson’s disease in mice via modulating gut microbiota and short-chain fatty acids
Source: Front Pharmacol. 2023 Jun 14;14:1198335. doi: 10.3389/fphar.2023.1198335 (PMC10303117; doi:10.3389/fphar.2023.1198335)

C MPTP Curcumin

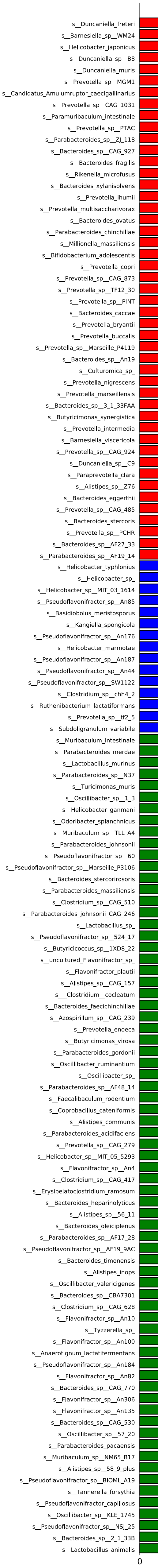

Supplement: Supplementary file 1 [file DataSheet2.PDF]

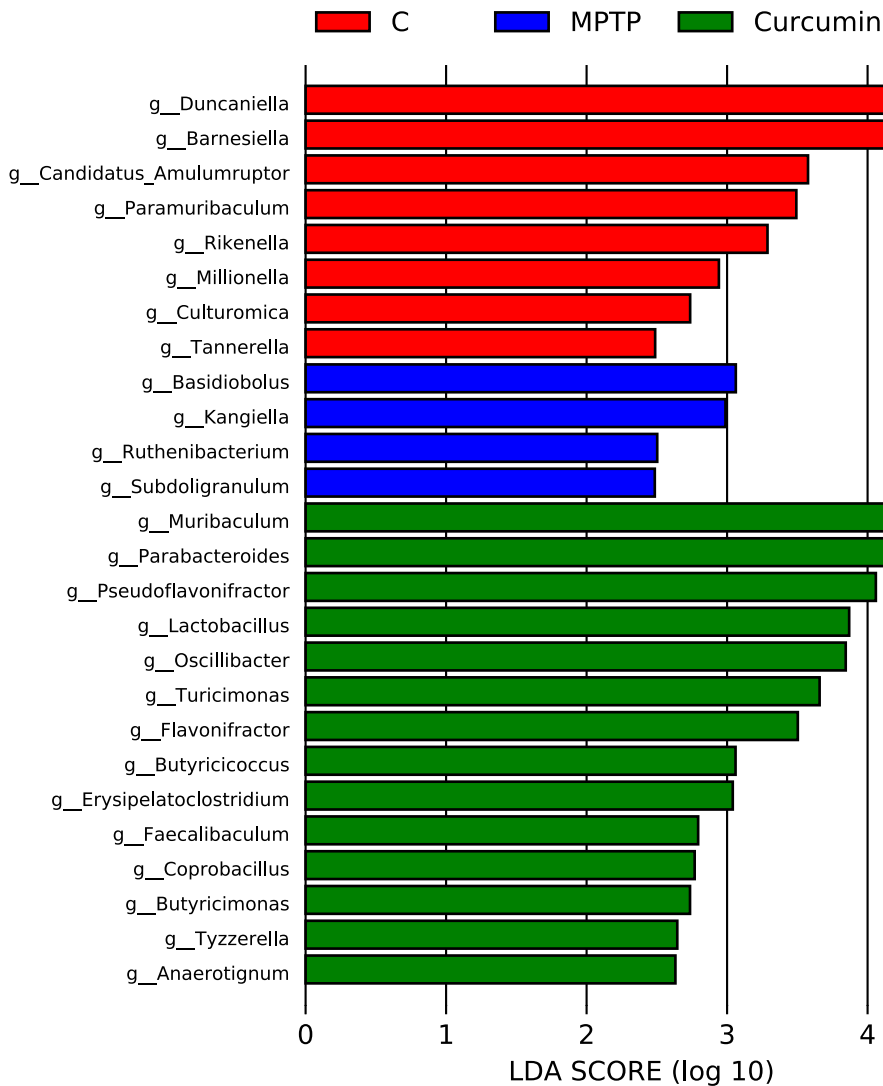

Supplement: Supplementary file 2 [file DataSheet1.PDF]
